# The use of novel diffuse optical spectroscopies for improved neuromonitoring during neonatal cardiac surgery requiring antegrade cerebral perfusion

**DOI:** 10.3389/fped.2023.1125985

**Published:** 2023-06-23

**Authors:** Kalil Shaw, Constantine D. Mavroudis, Tiffany S. Ko, Jharna Jahnavi, Marin Jacobwitz, Nicolina Ranieri, Rodrigo M. Forti, Richard W. Melchior, Wesley B. Baker, Arjun G. Yodh, Daniel J. Licht, Susan C. Nicolson, Jennifer M. Lynch

**Affiliations:** ^1^ Perelman School of Medicine, University of Pennsylvania, Philadelphia, PA, United States; ^2^ Division of Cardiothoracic Surgery, Children’s Hospital of Philadelphia, Philadelphia, PA, United States; ^3^ Department of Anesthesiology and Critical Care Medicine, Children’s Hospital of Philadelphia, Philadelphia, PA, United States; ^4^ Division of Neurology, Children’s Hospital of Philadelphia, Philadelphia, PA, United States; ^5^ Department of Perfusion Services, Children's Hospital of Philadelphia, Philadelphia, PA, United States; ^6^ Department of Physics and Astronomy, University of Pennsylvania, Philadelphia, PA, United States; ^7^ Division of Cardiothoracic Anesthesiology, Children’s Hospital of Philadelphia, Philadelphia, PA, United States

**Keywords:** neuromonitoring, optics, congenital heart diasease, antegrade cerebral perfusion, stage I Palliation-Norwood procedure, cerebral blood flow, hypoplastic left heart syndrome, NIRS (near infrared reflectance spectroscopy)

## Abstract

**Background:**

Surgical procedures involving the aortic arch present unique challenges to maintaining cerebral perfusion, and optimal neuroprotective strategies to prevent neurological injury during such high-risk procedures are not completely understood. The use of antegrade cerebral perfusion (ACP) has gained favor as a neuroprotective strategy over deep hypothermic circulatory arrest (DHCA) due to the ability to selectively perfuse the brain. Despite this theoretical advantage over DHCA, there has not been conclusive evidence that ACP is superior to DHCA. One potential reason for this is the incomplete understanding of ideal ACP flow rates to prevent both ischemia from underflowing and hyperemia and cerebral edema from overflowing. Critically, there are no continuous, noninvasive measurements of cerebral blood flow (CBF) and cerebral oxygenation (StO_2_) to guide ACP flow rates and help develop standard clinical practices. The purpose of this study is to demonstrate the feasibility of using noninvasive, diffuse optical spectroscopy measurements of CBF and cerebral oxygenation during the conduct of ACP in human neonates undergoing the Norwood procedure.

**Methods:**

Four neonates prenatally diagnosed with hypoplastic left heart syndrome (HLHS) or a similar variant underwent the Norwood procedure with continuous intraoperative monitoring of CBF and cerebral oxygen saturation (StO_2_) using two non-invasive optical techniques, namely diffuse correlation spectroscopy (DCS) and frequency-domain diffuse optical spectroscopy (FD-DOS). Changes in CBF and StO_2_ due to ACP were calculated by comparing these parameters during a stable 5 min period of ACP to the last 5 min of full-body CPB immediately prior to ACP initiation. Flow rates for ACP were left to the discretion of the surgeon and ranged from 30 to 50 ml/kg/min, and all subjects were cooled to 18°C prior to initiation of ACP.

**Results:**

During ACP, the continuous optical monitoring demonstrated a median (IQR) percent change in CBF of −43.4% (38.6) and a median (IQR) absolute change in StO_2_ of −3.6% (12.3) compared to a baseline period during full-body cardiopulmonary bypass (CPB). The four subjects demonstrated varying responses in StO_2_ due to ACP. ACP flow rates of 30 and 40 ml/kg/min (*n* = 3) were associated with decreased CBF during ACP compared to full-body CPB. Conversely, one subject with a higher flow6Di rate of 50 ml/kg/min demonstrated increased CBF and StO_2_ during ACP.

**Conclusions:**

This feasibility study demonstrates that novel diffuse optical technologies can be utilized for improved neuromonitoring in neonates undergoing cardiac surgery where ACP is utilized. Future studies are needed to correlate these findings with neurological outcomes to inform best practices during ACP in these high-risk neonates.

## Introduction

One in 100 neonates worldwide are born with congenital heart defects (CHD), a third of which require surgery or other interventions in early infancy (1, 2). Although advancements in the surgical and perioperative care of these neonates have improved survival rates over the last several decades, there remains a significant concern for neurodevelopmental disabilities (3–5). The highest risk cohort of patients are those who require neonatal interventions on the aortic arch and those with single ventricle physiology. While there are many reasons for these neonates to have worse neurological outcomes, one potentially modifiable risk factor is the choice of perfusion strategy during their neonatal cardiac surgery. Historically, the neuroprotective strategy of choice for neonatal aortic arch reconstruction has been deep hypothermic circulatory arrest (DHCA) where the patient is cooled to deep hypothermia (typically 18°C), exsanguinated, and the circulation stopped to permit intervention on the aortic arch in a bloodless field. However, recent reports have highlighted increased duration of DHCA as being associated with neurological injury (6–8). These associations have promoted the use of antegrade cerebral perfusion (ACP) where once the patient is cooled to deep hypothermia, corporeal circulation is arrested but unilateral flow is provided to the brain. Despite the hypothetical advantage of continued cerebral perfusion with ACP as compared to no cerebral perfusion during DHCA, there have been no conclusive clinical studies demonstrating the superiority of ACP over DHCA in short or long term neurological outcomes (9, 10).

Potential reasons for the lack of superiority of ACP over DHCA include an incomplete understanding of neurological injury following neonatal cardiac surgery and widely discrepant methods in the conduct of ACP among different institutions. Specifically, reports have shown ACP flow rates that vary from 20 to 94 ml/kg/min (11–13), and the use of neuromonitoring adjuncts such as near-infrared spectroscopy (NIRS), transcranial doppler (TCD), electroencephalography (EEG), or a combination of these modalities also vary widely (14). Moreover, because of the inherent limitations present in existing neuromonitoring techniques, there is no consensus regarding how best to tailor ACP flow rates to individual patients who may have vastly different perfusion requirements. Because of this, patients may be at risk for both ischemic and hyperemic injury during ACP that may have profound short- and long-term sequelae.

Conventional NIRS-based monitoring devices that are currently in use have been shown to be unreliable likely due to a series of assumptions that may limit their accuracy during deep hypothermia and during cardiopulmonary bypass (7). However, more sophisticated diffuse optical techniques exist that are well-suited for this clinical question. Frequency-domain diffuse optical spectroscopy (FD-DOS) differs from conventional NIRS in that it can *quantify* tissue concentration of oxy and deoxy-hemoglobin which allows for *quantification* of tissue oxygen saturation (15). Diffuse correlation spectroscopy (DCS) is a separate diffuse optical technique that employs near-infrared light to quantify tissue blood flow (16). Combined, FD-DOS and DCS permit noninvasive, continuous quantification of cerebral oxygen demand and utilization, which are the crucial parameters necessary for guiding ACP flow rate selection. Promising pilot data motivating the use of FD-DOS and DCS to guide ACP flow rate selection was previously shown in a neonatal swine model (17). The hybrid FD-DOS/DCS research device used in the present study has also been used in previous clinical studies (7, 18–21). The purpose of this study is to demonstrate the feasibility of using a hybrid FD-DOS/DCS optical instrument to monitor cerebral blood flow and oxygenation during ACP in neonates undergoing cardiac surgery.

## Methods

### Patient population

Term (37–42 weeks gestation) newborns with pre- or postnatally diagnosed hypoplastic left heart syndrome (HLHS) or variants admitted to the cardiac intensive care unit at the Children's Hospital of Philadelphia were screened for study inclusion and approached for participation as early as possible. Exclusion criteria included: birth weight <2 kg, a history of neonatal depression (e.g., 5 min APGAR <5, cord blood pH < 7.0, sepsis, or birth asphyxia), perinatal seizures, evidence of end-organ injury, preoperative cardiac arrest, and significant preoperative intracerebral hemorrhage such as grade 3 or 4 intraventricular hemorrhage. Infants with identified or suspected genetic syndromes were not excluded. Patient demographics are shown in Table 1.

**Table 1:** Patient demographics.

Subject	Gestational age (weeks)	Age at surgery (days)	Sex	Birth weight (kg)	Diagnosis
1	39.1	2	F	3.3	HLHS
2	39.1	3	F	3.2	HLHS
3	40.9	6	M	2.9	HLHS
4	38.0	5	F	2.6	Unbalanced right-sided CAVC, aortic arch hypoplasia

MA, mitral atresia; AA, aortic atresia; AS, aortic stenosis; CAVC, complete atrioventricular canal.

### Study protocol

The study protocol for this study was similar to a previously published study looking at cerebral physiology during DHCA (7). All procedures were approved by the Institutional Review Board at the Children's Hospital of Philadelphia. Patient demographic data were recorded. On the morning of surgery and after induction of general anesthesia, a noninvasive optical probe was secured to the forehead for continuous optical measurements of cerebral physiology. All patients underwent cardiac surgery utilizing cardiopulmonary bypass with antegrade cerebral perfusion (ACP). The ACP flow rate used for each case was determined by the preferred flow rate of the surgeon assigned to the case, with values of 30 ml/kg/min (*n* = 1), 40 ml/kg/min (*n* = 2), and 50 ml/kg/min (*n* = 1). ACP flow rates were not adjusted throughout the ACP period and the flow rate choice was not influenced by this study. ACP was delivered either through an expanded polytetrafluorethylene shunt sewn to the innominate artery (*n* = 3), or via direct cannulation of the innominate artery (*n* = 1) with occlusion of the innominate artery proximal to the cannulation site for selective ACP administration. pH- stat blood gas management was used during cooling and while hypothermic; alpha stat was used during rewarming and at normothermia per institutional protocol. Patients were cooled to a target nasopharyngeal (NP) temperature of 18°C prior to initiating ACP. FD-DOS and DCS data were collected continuously but were not disclosed to the clinical team and were not used to guide any clinical management.

### Hybrid FD-DOS/DCS measurements

This study employs a hybrid optical research device combining two techniques: frequency-domain diffuse optical spectroscopy (FD-DOS) and diffuse correlation spectroscopy (DCS). These techniques, which utilize near-infrared light to noninvasively probe static and dynamic physiological properties of cortical brain tissue, have been described in depth previously (7, 19, 20).

Multi-distance FD-DOS is capable of accurate quantification of cerebral tissue oxygen saturation (StO_2_), as opposed to commercial oximeters that employ continuous-wave NIRS (CW-NIRS or NIRS) to monitor trends in cerebral oxygen saturation. FD-DOS uses photon diffusion theory to relate the measured amplitude attenuation and phase shift of modulated and multiply scattered light detected on the tissue surface to determine the wavelength-dependent tissue absorption (µ_a_) and scattering (µ'_s_) properties. These properties are determined by fitting to the semi-infinite medium solution of the photon diffusion equation for a homogeneous medium. The phase and AC amplitude (AC) of the detected light quantify the optical properties of the tissue. Specifically, the slope of the phase vs. source-detector separation on the tissue surface (*r*) and the slope of the ln(AC ×  *r*2) vs. *r* were determined and used to compute µ_a_ and µ'_s_, respectively. Data were discarded if the linear fits had a Pearson correlation coefficient *R*^2^ < 0.975, which was usually caused by probe dislodgement during patient repositioning.

The wavelength and time-dependent absorption coefficient, µ_a_(*λ,t*), in turn, depends linearly on oxy- [(HbO_2_)] and deoxy-hemoglobin [(Hb)] concentration. Therefore, measurements of the tissue absorption at multiple wavelengths yield these 2 concentrations in absolute units. From [HbO_2_] and [Hb], we also derived total hemoglobin concentration (THC = [HbO_2_] + [Hb]) and cerebral tissue oxygen saturation [StO_2 _= (HbO_2_)/THC]. The FD-DOS device employed in the present study (Imagent, ISS Inc., Champaign, IL) is amplitude modulated at 110 MHz and employs source lasers at 4 wavelengths, *λ* = 688, 725, 785 and 830 nm.

Diffuse correlation spectroscopy (DCS) uses near-infrared light to noninvasively monitor cerebral blood flow (CBF). DCS measures the temporal fluctuations (the temporal autocorrelation function) of the light intensity at the tissue surface which is primarily caused by moving red blood cells. The correlation diffusion equation and its semi-infinite solutions were employed along with the tissue optical absorption and scattering measured in real-time with FD-DOS to convert these temporal fluctuations into an index of cerebral blood flow (CBF_i_, measured in units of cm^2^/s). Although this index does not have traditional physiological units of CBF, recent studies have shown that CBF_i_ can be calibrated in absolute units, and that it correlates strongly with other gold standard measures of CBF (23, 24). Jain et al., for example, validated this optically measured CBF_i_ against CBF measured in the superior sagittal sinus with phase contrast MRI in a similar population of infants with critical CHD (25). DCS has also been shown to correlate with TCD measurements of CBF (26), even though DCS is measuring blood flow in the tissue microvasculature whereas TCD measures flow in cerebral arteries.

The patient interface for this hybrid FD-DOS/DCS instrument consisted of a custom-made flexible black rubber probe secured to the subject's forehead with a soft wrap. The probe houses fiber optics for both FD-DOS and DCS. For FD-DOS, we used 4 source-detector separations (1.5, 2.0, 2.5, and 3.0 cm along the tissue surface), with 4 source fibers (i.e., one for each wavelength at each separation) and 1 detection fiber. For DCS, 8 single-mode detection fibers were bundled and placed on the tissue surface 2.5 cm away from the source fiber.

The change in StO_2_ due to ACP was calculated by comparing StO_2_ during a stable 5 min window during ACP to a 5 min baseline period during full-body CPB immediately prior to the initiation of ACP using the following formula:

$$\Delta StO_{2}\equiv StO_{2,\;ACP}-StO_{2,CPB}$$

The change in CBF due to ACP was calculated comparing the same time windows as above using the following formula:

$$rCBF\equiv \frac{CBF_{ACP}-CBF_{CPB}}{CBF_{CPB}}\;\times \;100\text{\%}$$

## Results

**Figure 1:**
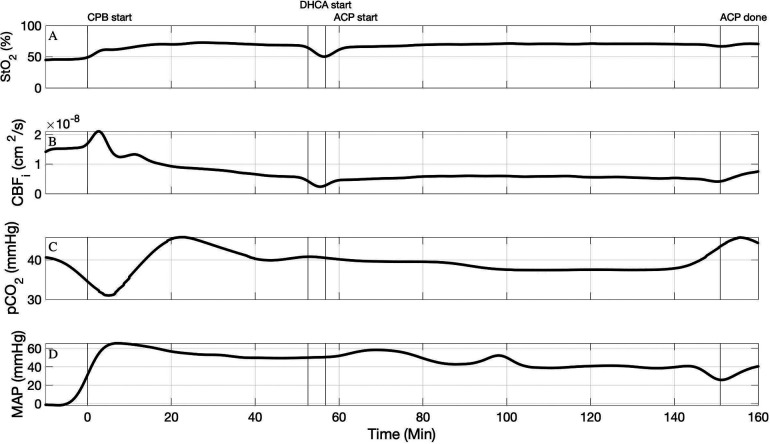
Timeseries of FD-DOS measured StO_2_ (**A**), DCS measured CBF_i_ (**B**), pCO_2_ (corrected for 37°C) (**C**), and mean arterial blood pressure (**D**) in subject #3. An ACP flow rate of 30 ml/kg/min was used.

Our hybrid FD-DOS/DCS device was used for continuous intraoperative monitoring in four neonates with HLHS or similar variants during the Norwood procedure. Table 2 lists the changes in StO_2_ and CBF_i_ due to ACP compared to CPB for all four subjects. We observed a decrease in CBF_i_ during ACP in 3 of the 4 patients with ACP flow rates of 30 and 40 ml/kg/min. Figure 1 demonstrates the timeseries for StO_2_ and CBF_i_ for Subject #3 where the ACP flow rate was 30 ml/kg/min. In this subject, CBF_i_ is lower during ACP than it was during full-body CPB, however StO_2_ does not significantly change which suggests that cerebral oxygen demand is being met despite the decrease in cerebral oxygen delivery.

**Table 2:** ACP flow rate, CPB and ACP durations, and changes in StO_2_ and CBF due to ACP for each of the four subjects.

Subject	ACP flow rate (ml/kg/min)	CPB duration (min)	ACP duration (min)	ΔStO_2_ (%)	rCBF (%)
1	40	55	45	−3.8 ± 2.3	−29.2 ± 17.6
2	50	36	75	13.3 ± 4.0	156.3 ± 103.9
3	30	55	94	1.2 ± 3.0	−13.5 ± 9.4
4	40	47	55	−7.0 ± 4.6	−53.1 ± 47.5

**Figure 2:**
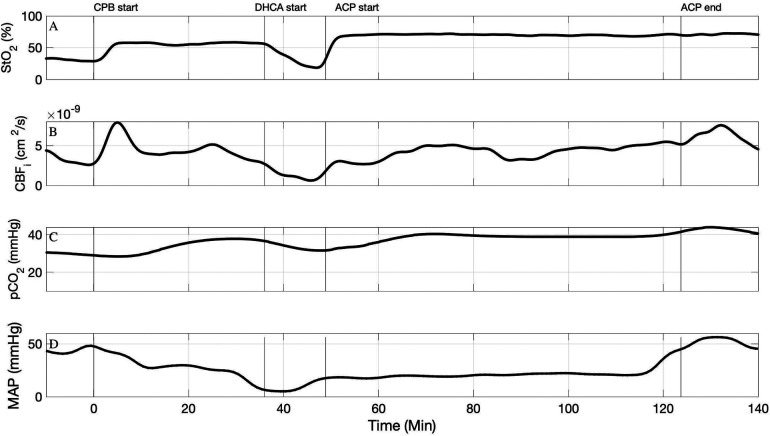
Timeseries of FD-DOS measured StO_2_ (**A**), DCS measured CBF_i_ (**B**), pCO_2_ (corrected for 37°C) (**C**), and mean arterial blood pressure (**D**) in subject #2. An ACP flow rate of 50 ml/kg/min was used.

Figure 2 demonstrate the timeseries for StO_2_ and CBF_i_ for Subject #2 where the ACP flow rate was 50 ml/kg/min. In this subject, we observed an increase in CBF_i_ during ACP compared to the baseline period on full-body CPB. Interestingly in this subject we also observed an increase in StO_2_ from the period of full-body bypass to ACP, suggesting that the cerebral oxygen delivery was exceeding cerebral oxygen demand.

## Discussion

In this report, we demonstrate the first use of hybrid FD-DOS/DCS for continuous, noninvasive cerebral monitoring in four neonates undergoing cardiac surgery requiring ACP. We observed different trends in cerebral oxygen saturation and blood flow among the four subjects. Perhaps unsurprisingly, the one subject with the highest ACP flow rate experienced higher CBF and StO_2_ during ACP compared to baseline, while the subjects with lower ACP flow rate experienced varying degrees of decreased CBF and less change in StO_2_ compared to the baseline full-body CPB period. While this sample size is too small to derive any conclusions on optimal flow rate, these cases demonstrate the variance in cerebral hemodynamic response to various ACP flow rates among different neonates as well as the feasibility to quantify these changes non-invasively with hybrid FD-DOS/DCS.

The lack of accurate neuromonitoring during neonatal cardiac surgery, specifically during procedures where ACP and DHCA are employed, leads to numerous and critical gaps in the understanding of the neonatal brain's metabolic and physiologic needs at such extremes of cardiovascular physiology. This issue is further exacerbated by widely disparate practices concerning the conduct of cardiopulmonary bypass, flow rates during ACP, operative and perioperative anesthetic regimens that may affect cerebrovascular reactivity, temperature at which ACP is initiated, duration of cooling and rewarming before and after ACP, and many other patient-specific factors that can lead to different cerebral metabolic needs and responses to cardiopulmonary bypass. Current neuromonitoring techniques have failed to improve neurodevelopmental outcomes according to a recent systemic review (27), which belies an incomplete understanding of how best to match cerebral metabolic demand of neonatal with cerebral oxygen delivery in neonatal cardiac patients. While some groups have described trends in decreasing neurological injury using higher ACP flow rates and multimodal neuromonitoring strategies that employ conventional NIRS with TCD to assess CBF (14), these neuromonitoring modalities have several limitations. The unreliability of TCD was demonstrated in a study by Hofer et al. where 10 neonates undergoing the Norwood procedure were monitored with bilateral cerebral NIRS and TCD (28). In this study, NIRS monitoring was deemed reliable in all 10 subjects, but TCD was unreliable 10%–40% of the time depending on ACP flow rate. However, as mentioned previously, conventional NIRS only allows for trend monitoring of cerebral saturation due to the assumption that must be made of the scattering property of tissue. Even as a trend monitor, conventional NIRS appears to have limitations at extreme physiologic states (7). Furthermore, employing only NIRS does not offer information about perfusion, and a change in cerebral saturation measured with NIRS could be incorrectly attributed to a change in perfusion instead of a change in oxygen extraction or vice versa. TCD offers information on perfusion, but continuous monitoring with TCD is challenging due to difficulty ensuring stable fixation of the TCD probe. Furthermore, TCD probes large arteries whereas conventional NIRS probes the microvasculature, which makes it difficult to interpret these data together. Hybrid FD-DOS/DCS offers a noninvasive way to continuously probe the same microvasculature for quantification of both oxygen delivery and oxygen utilization.

While the use of hybrid FD-DOS/DCS provides more precise and potentially useful data than existing neuromonitoring strategies, there are also limitations to its use that are important to discuss. Firstly, these data are experimental, and critical values that warrant investigation or intervention have not yet been established. The issue of critical values for intervention has also not yet been resolved with conventional NIRS and TCD; while some centers have proposed such criteria (14), there is no consensus concerning how best to use conventional NIRS with or without TCD to monitor cerebral oxygen delivery during ACP. As our experimental and clinical experience with FD-DOS/DCS increases, we hope to address this major shortcoming and to develop clinical protocols that can best leverage this technology as both a diagnostic and a therapeutic monitoring device. These data have also not yet been correlated with clinical or radiographical evidence of neurological injury from ACP. We have previously correlated FD-DOS measured changes in cerebral oxygen saturation during DHCA with white matter injury on post-operative MRI in this same patient population (7) and plan to investigate this further in patients where ACP is used.

Despite the inherent limitations associated with novel technology in its infancy, the ability to obtain absolute measurements of key neurovascular and neurometabolic indices without technological limitations or assumptions represents a very promising breakthrough in neuromonitoring. By overcoming many of the shortcomings of existing neuromonitoring technologies, hybrid FD-DOS/DCS may prove invaluable as a way to match cerebral perfusion to metabolic demand. Matching supply with demand has the most direct effect on optimizing ACP delivery during neonatal cardiac surgery, but this technology may also be applicable to other disease states where perfusion to different organ beds may be compromised.

## Data Availability

The original contributions presented in the study are included in the article, further inquiries can be directed to the corresponding author.

## References

[1] Hoffman JIE Kaplan S. The incidence of congenital heart disease. J Am Coll Cardiol. (2002) 39(12):1890–900. 10.1016/S0735-1097(02)01886-7 12084585

[2] Wu W He J Shao X. Incidence and mortality trend of congenital heart disease at the global, regional, and national level, 1990-2017. Medicine. (2020) 99(23):e20593. 10.1097/MD.0000000000020593 PMC7306355 32502030

[3] Mahle WT Clancy RR Moss EM Gerdes M Jobes DR Wernovsky G. Neurodevelopmental outcome and lifestyle assessment in school-aged and adolescent children with hypoplastic left heart syndrome. Pediatrics. (2000) 105(5):1082–9. 10.1542/peds.105.5.1082 10790466

[4] Marino BS Lipkin PH Newburger JW Peacock G Gerdes M Gaynor JW Neurodevelopmental outcomes in children with congenital heart disease: evaluation and management: a scientific statement from the American heart association. Circulation. (2012) 126(9):1143–72. 10.1161/CIR.0b013e318265ee8a 22851541

[5] Lynch JM Gaynor JW Licht DJ. Brain injury during transition in the newborn with congenital heart disease: hazards of the preoperative period. Semin Pediatr Neurol. (2018) 28:60–5. 10.1016/j.spen.2018.05.007 30522729

[6] Beca J Gunn JK Coleman L Hope A Reed PW Hunt RW New white matter injury after infant heart surgery is associated with diagnostic group and use of circulatory arrest. Circulation. (2013) 127:917–79. 10.1161/CIRCULATIONAHA.112.001089 23371931

[7] Lynch JM Mavroudis CD Ko TS Jacobwitz M Busch DR Xiao R Association of ongoing cerebral oxygen extraction during deep hypothermic circulatory arrest with post-operative brain injury. Semin Thorac Cardiovasc Surg. (2022) 34(4):1275–84. 10.1053/j.semtcvs.2021.08.026 34508811 PMC8901799

[8] Bellinger DC Wypij D duPlessis AJ Rapaport LA Jonas RA Wernovsky G Neurodevelopmental status at eight years in children with dextro-transposition of the great arteries: the Boston circulatory arrest trial. J Thorac Cardiovasc Surg. (2003) 126(5):1385–96. 10.1016/S0022-5223(03)00711-6 14666010

[9] Algra SO Jansen NJG van der Tweel I Schouten ANJ Groenendaal F Toet M Neurological injury after neonatal cardiac surgery: a randomized controlled trial of two perfusion techniques. Circulation. (2014) 129(2):224–33. 10.1161/CIRCULATIONAHA.113.003312 24141323

[10] Goldberg CS Bove EL Devaney EJ Mollen E Schwartz E Tindall S A randomized clinical trial of regional cerebral perfusion versus deep hypothermic circulatory arrest: outcomes for infants with functional single ventricle. J Thorac Cardiovasc Surg. (2007) 133(4):880–7. 10.1016/j.jtcvs.2006.11.029 17382619

[11] Andropoulos DB Stayer SA McKenzie ED Fraser CD. Regional low-flow perfusion provides comparable blood flow and oxygenation to both cerebral hemispheres during neonatal aortic arch reconstruction. J Thorac Cardiovasc Surg. (2003) 126(6):1712–7. 10.1016/S0022-5223(03)01027-4 14688677

[12] Andropoulos DB Stayer SA McKenzie ED Fraser CD Pigula FA Austin EH. Novel cerebral physiologic monitoring to guide low-flow cerebral perfusion during neonatal aortic arch reconstruction. J Thorac Cardiovasc Surg. (2003) 125(3):491–9. 10.1067/mtc.2003.159 12658190

[13] McQuillen PS Barkovich AJ Hamrick SEG Perez M Ward P Glidden DV Temporal and anatomic risk profile of brain injury with neonatal repair of congenital heart defects. Stroke. (2007) 38(2 Suppl):736–41. 10.1161/01.STR.0000247941.41234.90 17261728

[14] Fraser CD Andropoulos DB. Principles of antegrade cerebral perfusion during arch reconstruction in newborns/infants. Semin Thorac Cardiovasc Surg Pediatr Card Surg Annu. (2008) 11(1):61–8. 10.1053/j.pcsu.2007.12.005 PMC2531241 18396227

[15] Durduran T Choe R Baker WB Yodh AG. Diffuse optics for tissue monitoring and tomography. Reports Prog Phys. (2010) 73:076701. 10.1088/0034-4885/73/7/076701 PMC4482362 26120204

[16] Durduran T Yodh AG. Diffuse correlation spectroscopy for non-invasive, micro-vascular cerebral blood flow measurement. Neuroimage. (2014) 85(1):51–63. 10.1016/j.neuroimage.2013.06.017 23770408 PMC3991554

[17] Mavroudis CD Ko T Volk LE Smood B Morgan RW Lynch JM Does supply meet demand? A comparison of perfusion strategies on cerebral metabolism in a neonatal swine model. J Thorac Cardiovasc Surg. (2022) 163:e47–58. 10.1016/j.jtcvs.2020.12.005 33485668 PMC8862716

[18] Lynch JM Buckley EM Schwab PJ McCarthy AL Winters ME Busch DR Time to surgery and preoperative cerebral hemodynamics predict postoperative white matter injury in neonates with hypoplastic left heart syndrome. J Thorac Cardiovasc Surg. (2014) 148(5):P2181–8. 10.1016/j.jtcvs.2014.05.081 PMC4254035 25109755

[19] Lynch JM Ko T Busch DR Newland JJ Winters ME Mensah-Brown K Preoperative cerebral hemodynamics from birth to surgery in neonates with critical congenital heart disease. J Thorac Cardiovasc Surg. (2018) 156(4):1657–64. 10.1016/j.jtcvs.2018.04.098 PMC6166233 29859676

[20] Durduran T Zhou C Buckley EM Kim MN Yu G Choe R Optical measurement of cerebral hemodynamics and oxygen metabolism in neonates with congenital heart defects. J Biomed Opt. (2010) 15(3):37004. 10.1117/1.3425884 PMC2887915 20615033

[21] Buckley EM Lynch JM Goff DA Schwab PJ Baker WB Durduran T Early post-operative changes in cerebral oxygen metabolism following neonatal cardiac surgery: effects of surgical duration. J Thorac Cardiovasc Surg. (2012) 145(1):196–205. 10.1016/j.jtcvs.2012.09.057 23111021 PMC3658109

[22] Ayaz H Baker WB Glaney G Boas DA Bortfeld H Brady K Optical imaging and spectroscopy for the study of the human brain: status report. Neurophotonics. (2022) 9(S2):1–65. 10.1117/1.NPh.9.S2.S24001 PMC9424749 36052058

[23] Buckley EM Hance D Pawlowski T Lynch J Wilson FB Mesquita RC Validation of diffuse correlation spectroscopic measurement of cerebral blood flow using phase-encoded velocity mapping magnetic resonance imaging. J Biomed Opt. (2012) 17(3):037007. 10.1117/1.JBO.17.3.037007 22502579 PMC3380925

[24] Giovannella M Andersen B Andersen JB El-Mahdaoui S Contini D Spinelli L Validation of diffuse correlation spectroscopy against 15O-water PET for regional cerebral blood flow measurement in neonatal piglets. J Cereb Blood Flow Metab. (2020) 40(10):2055–65. 10.1177/0271678X19883751 31665953 PMC7786848

[25] Jain V Buckley EM Licht DJ Lynch JM Schwab PJ Naim MY Cerebral oxygen metabolism in neonates with congenital heart disease quantified by MRI and optics. J Cereb Blood Flow Metab. (2014) 34(3):380–8. 10.1038/jcbfm.2013.214 24326385 PMC3948119

[26] Buckley EM Cook NM Durduran T Kim MN Zhou C Choe R Cerebral hemodynamics in preterm infants during positional intervention measured with diffuse correlation spectroscopy and transcranial Doppler ultrasound. Opt Express. (2009) 17(15):12571–81. 10.1364/OE.17.012571 19654660 PMC2723781

[27] Hirsch JC Jacobs ML Andropoulos D Austin EH Jacobs JP Licht DJ Protecting the infant brain during cardiac surgery: a systematic review. Ann Thorac Surg. (2012) 94:1365–73. 10.1016/j.athoracsur.2012.05.135 PMC4249676 23006704

[28] Hofer A Haizinger B Geiselseder R Mair R Rehak P Gombotz H. Monitoring of selective antegrade cerebral perfusion using near infrared spectroscopy in neonatal aortic arch surgery. Eur J Anaesthesiol. (2005) 22(4):293–8. 10.1017/S0265021505000499 15892408

